# Unlocking the Potential of Thermal Post-Treatments: A Study on Odor Emission Control in *Eucalyptus* Wood Particleboard

**DOI:** 10.3390/molecules30091949

**Published:** 2025-04-28

**Authors:** Wenhang Yin, Yueyun Zhang, Churan Li, Boxiao Wu, Zhaojin Yang, Heming Huang, Bangrui Luo, Guanben Du, Ping Zhao, Xiaoqin Yang

**Affiliations:** 1 Key Laboratory of National Forestry and Grassland Administration on Highly-Efficient Utilization of Forestry Biomass Resources in Southwest China, Southwest Forestry University, Kunming 650224, China; ywh123@swfu.edu.cn (W.Y.); m15398742192@163.com (Y.Z.); churanli@swfu.edu.cn (C.L.); wbx1437@swfu.edu.cn (B.W.); guanben@swfu.edu.cn (G.D.); 2 Kunming Feilin Wood-Based Panel Group Co., Ltd., Kunming 650033, China; 13888960633@163.com (Z.Y.); 13888443607@163.com (H.H.); 13888619117@163.com (B.L.); 3 Key Laboratory of Ministry of Education for Forest Resources Conservation and Utilization in the Southwest Mountains of China, Southwest Forestry University, Kunming 650224, China

**Keywords:** *Eucalyptus* wood particleboard, volatile organic compound, thermal post-treatments, headspace solid-phase microextraction, key odorant compounds, indoor air quality

## Abstract

*Eucalyptus* wood particleboard (EPB), commonly used in indoor decoration, releases volatile organic compounds (VOCs) that can adversely affect indoor air quality and human health. This study systematically examined the VOC emission characteristics of EPB using headspace solid-phase microextraction (HS-SPME) coupled with gas chromatography mass spectrometry (GC-MS). A total of 65 VOCs were identified, with medium-volatility organic compounds (MVOCs) accounting for 28 compounds, low-volatility organic compounds (LVOCs) for 26, and high-volatility organic compounds (HVOCs) for 11. Terpenoids dominated the VOCs, comprising 78.46%, followed by aldehydes (10.77%) and alkanes (7.69%). Key odorant compounds (KOCs) were identified using the relative odor activity value (ROAV), with hexanal (ROAV = 100) and o-cymene (ROAV = 76.90) emerging as the most significant contributors to the overall odor profile. Thermal post-treatment at temperatures of 50–60 °C for durations of 6–12 h was found to be an effective method for reducing the residual VOCs and KOCs in the EPB, leading to a marked decrease in the peak areas of key odorants. The findings suggest several strategies for minimizing VOC emissions and eliminating residual odor, including reducing the use of miscellaneous wood materials, controlling the production of o-cymene, and employing thermal post-treatment at moderate temperatures. These measures provide a promising approach to reducing VOC and odor emissions from EPB and similar composite wood products, thereby enhancing their suitability for indoor applications. This study innovatively establishes an evaluation system for VOC emission characteristics in wood-based panels based on the ROAV. It elucidates the contribution mechanisms of key odor-active substances (e.g., hexanal and pentanal) and presents a thermal post-treatment process for source control, achieving simultaneous VOCs and odor elimination. A ROAV-guided hierarchical management strategy is proposed, providing scientific guidelines for the industrial production of high-quality particleboards with ultralow emissions (TVOC < 50 μg/m^3^) and minimal odor intensity (OI < Grade 3).

## 1. Introduction

With global population growth and economic development, the overexploitation of forest resources has caused severe timber shortages, driving the search for alternative materials [1,2,3]. Particleboard, also known as chipboard, is a key engineered wood product made by processing wood or other lignocellulosic materials into small particles, which are then dried, bonded with adhesives, and pressed into panels under heat and pressure. Its production utilizes sustainable resources such as thinning wood, small-diameter timber, logging residues, agricultural by-products, and wood or bamboo waste, enhancing resource efficiency and reducing dependence on natural forests [4,5]. With a uniform structure, excellent workability, and properties like sound and thermal insulation, particleboard is widely used in furniture, interior decoration, and construction [6,7]. However, raw material selection significantly impacts its quality, cost, and performance. Low-density materials like straw can reduce strength, while high-moisture materials complicate drying. Transportation costs also challenge scalability, especially when raw materials must be hauled over long distances [8,9]. Fast-growing forests offer a viable solution. Species like *Eucalyptus* and *Poplar*, with rapid growth cycles and high yields, ensure a stable raw material supply, reduce transport costs, and meet production stability requirements. Their moderate density and plasticity make them ideal for particleboard production, alleviating timber shortages and supporting sustainable industry growth [10,11,12].

*Eucalyptus* species are key members of fast-growing artificial forests, characterized by their wide distribution, resource abundance, resistance to adverse conditions, short harvesting cycle, and low production costs, making *Eucalyptus* wood an ideal raw material for particleboard. However, despite undergoing high-temperature pressing at 245 °C, *Eucalyptus* wood particleboard (EPB) often emits a strong, pungent odor, which compromises its suitability as an indoor material. In preliminary studies, headspace solid-phase microextraction (HS-SPME) coupled with gas chromatography mass spectrometry (GC-MS) was used to analyze the volatile organic compounds (VOCs) released from *Eucalyptus* wood raw materials, dry surface shavings, dry core shavings, and finished particleboard. The results indicated that terpenoids and other volatile substances in *Eucalyptus* wood are the primary sources of these VOCs [13]. While such compounds can be beneficial in certain applications, like fragrances, their strong odor makes EPB less suitable for indoor environments, where VOCs pose significant health risks, including respiratory issues and sick building syndrome (SBS) [14,15,16,17].

The release of VOCs from building materials is a growing concern, with over 200 VOCs identified in indoor environments [18,19,20]. Prolonged exposure to low-level VOCs can lead to neurotoxic effects, including symptoms such as irritation, respiratory infections, and asthma [21,22,23]. Source control is the most effective strategy for improving indoor air quality. Therefore, studying VOC emission control in engineered wood products, particularly to develop low-emission boards, is critical for enhancing indoor air quality. Recent research has focused on VOC emissions and their control in engineered wood products. An et al. (2011) found that the release of total VOCs and formaldehyde from wood flooring was linked to heating systems, with underfloor heating increasing formaldehyde emissions compared to air circulation systems [24]. In 2016, Li Dandan explored the impact of heating treatment on VOC emissions from particleboard and developed a catalytic material to remove formaldehyde [25]. Ayrilmis (2016) showed that using nanocellulose in urea–formaldehyde resins reduced total VOC and formaldehyde emissions from wood-based panels [26]. Other studies have also demonstrated that heat treatment can effectively control VOC emissions [27,28,29,30,31,32,33]. Previous studies have predominantly focused on the mechanical properties and formaldehyde emissions of particleboards, while limited attention has been paid to the systematic investigation of VOCs and the release profiles of characteristic odorants. With growing consumer demands for environmental sustainability and safety in building materials, developing particleboards with low VOC emissions is critical to enhancing market competitiveness. This study aims to achieve the sustained mitigation of VOCs and associated odor-active compounds at the manufacturing stage through raw material optimization and process refinement, thereby minimizing potential health risks in end-use scenarios. These findings suggest that heat treatment, particularly post-pressing heat treatment, could significantly reduce VOC emissions and improve odor characteristics in EPB. However, the effectiveness of heat treatment depends on factors such as treatment temperature, duration, and the chemical composition of the wood. Optimizing the heat treatment process is crucial to mitigating off odors and enhancing the suitability of *Eucalyptus* particleboard for indoor use. This study aims to optimize the post-heat treatment process for EPB by analyzing VOCs through HS-SPME coupled with GC-MS, identifying key odor components, and reducing harmful emissions. The findings will offer valuable insights into addressing odor issues and support the development of sustainable, low-emission products, contributing to the healthier growth of the industry.

Current research predominantly focuses on regulating VOC emissions through manufacturing parameters (e.g., hot-pressing temperature, duration, resin ratio), achieving progress in total VOC quantification while neglecting odor-centric analyses, particularly dynamic assessments of odor intensity. This study transcends conventional passive monitoring paradigms by introducing an odor OAV-based quantitative framework, synergistically integrating thermal post-processing optimization and sustainable manufacturing protocols to establish a holistic solution for proactive odor source elimination and process enhancement. These innovations address critical gaps in olfactory pollution control mechanisms and align with global demands for eco-friendly building materials, offering implementable strategies to advance low-carbon transitions in the construction industry.

## 2. Results

### 2.1. Identification of VOCs

**Figure 1:**
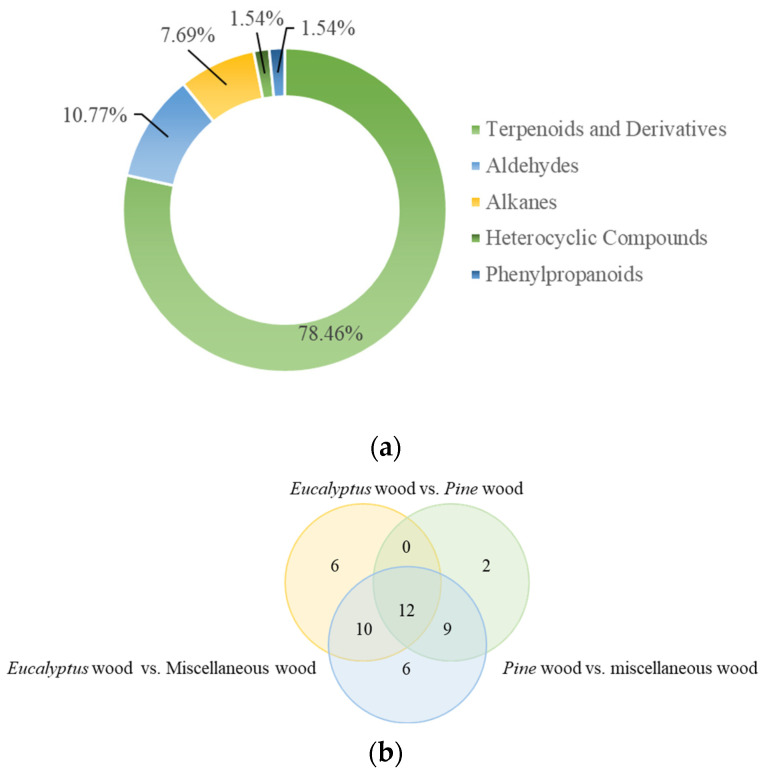
VOCs detected in *Eucalyptus* particleboard: (**a**) classification and (**b**) Venn diagram.

The concentration and composition of VOCs are crucial to the olfactory perception of EPB, directly influencing consumer acceptance [34]. Using HS-SPME coupled with GC-MS, the emission characteristics of VOCs were analyzed, as shown in Figure 1a and detailed in Table S1. A total of 65 VOCs were identified. Analysis of the boiling points of these compounds revealed that medium-volatility organic compounds (MVOCs) predominated, accounting for 28 compounds. Low-volatility organic compounds (LVOCs) were represented by 26 compounds, while high-volatility organic compounds (HVOCs) were limited to 11. This distribution indicates that MVOCs were the dominant VOCs detected. Their prevalence may be due to their ability to partially volatilize while remaining in significant proportions during the high-temperature pressing process, making them key components of VOC emissions. In contrast, HVOCs, with higher volatility, were likely released during pressing and thus detected in lower quantities in the final particleboard. LVOCs, due to their structural stability and lower volatility, were better retained under high-temperature conditions, resulting in their prominent presence in the particleboard. This volatility distribution reflects both the production process and the inherent stability of the compounds. It has important implications for the particleboard’s olfactory properties and its environmental impact, particularly concerning indoor air quality and consumer health [21,22,23].

As shown in Figure 1a, the 65 VOCs detected in EPB were classified into seven categories based on their molecular structure. Terpenoids and derivatives accounted for the majority, with 51 compounds (78.46%), followed by aldehydes (7 compounds, 10.77%), alkanes (5 compounds, 7.69%), 1 heterocyclic compound (1.54%), and 1 phenylpropanoid (1.54%). This distribution highlights the dominance of terpenes and terpenoids, which are well-known for their aromatic properties and biological activities, significantly influencing both the olfactory and biological characteristics of EPB. Aldehydes primarily contribute fatty, green, and fruity aromas. Aldehydes, including octanal, nonanal, hexanal, heptanal, furfural, decanal, and 2-methyl-3-phenylpropanal, were detected with a relative content of 3.35%. Among them, hexanal accounted for 1.67%, characterized by a green, grassy, and fresh scent. Due to its low odor threshold, hexanal is often considered a source of pungent odors [35]. Furfural, with a relative content of 0.55%, has a notably low OT. Among the seven compounds contributing to astringency, furfural ranked second to last in terms of astringency threshold. Among the five compounds imparting bitterness, furfural had the lowest bitterness threshold, indicating that even trace amounts can produce strong burnt, bitter, and astringent sensory perceptions. Its sensory profile is described as intensely pungent and bitter when concentrated, with a faint cinnamon oil aroma at low concentrations. For alkanes, compounds such as tricosane, tetracosane, heneicosane, eicosane, and docosane were identified with a relative content of 2.22%. Alkanes typically have high odor thresholds, resulting in a minimal impact on aroma. The heterocyclic compound 2-pentylfuran, with a relative content of 0.88%, exhibits distinctive green, beany, and grassy scents, making it a valuable ingredient for food flavor formulations. Among phenylpropanoids, safrole was detected at a relative content of 0.07%. Safrole has a spicy, camphor-like aroma and is commonly used as a food additive [36]. As detailed in Table S1, the most abundant VOC identified was aromadendrene, comprising approximately 24% of the total VOCs. This sesquiterpenoid is characterized by a moderately woody, resinous, and earthy aroma, along with notable biological activities, including antibacterial, anti-inflammatory, antidepressant, and anticancer effects [37,38]. The second most abundant compound was viridiflorol, another sesquiterpenoid, accounting for about 16% of the VOCs. Known for its rich woody, floral, and herbal fragrance, it is widely used as a fragrance, flavoring agent, and cosmetic ingredient, enhancing aroma and taste across various applications [39,40]. The third most abundant compound, ledene, also a sesquiterpenoid, constituted approximately 8% of the VOCs. It is distinguished by its woody, herbal, and green scent, making it a valuable fixative in the perfume industry, with additional antimicrobial and anti-inflammatory properties that are important for cosmetics and pharmaceuticals [41].

Although these naturally derived compounds are generally considered safe at appropriate concentrations, exposure to high levels or prolonged contact, especially under elevated temperatures, may lead to skin or mucous membrane irritation [21,22,23]. Additionally, 25 terpenoids and derivatives were classified as MVOCs, 22 as LVOCs, and 5 as HVOCs. The predominance of MVOCs and LVOCs suggests a balance between sufficient volatility to contribute to olfactory perception and retention within the EPB matrix. However, HVOCs may accumulate in poorly ventilated indoor environments, potentially degrading air quality and posing health risks [34,35].

The source analysis of the compounds (Table S1) indicates that *Eucalyptus* wood, *Pine* wood, and miscellaneous wood are the primary contributors to the VOCs in EPB. Specifically, 45 compounds, including 36 terpenoids and derivatives, 6 aldehydes, 2 alkanes, and 1 furan, were derived from the wood materials. In contrast, three alkanes, including tetracosane, eicosane, and heneicosane, were only detected in the additives. Notably, 17 compounds, including 15 terpenes and terpenoids, 1 phenylpropanoid, and 1 aldehyde, were not detected in the raw materials or additives but were found in the final EPB. This suggests the occurrence of new chemical reactions during the board production process, likely triggered by heat-press treatment, which led to the formation of new VOCs. Furthermore, the low concentration of some compounds in the raw materials and additives may have been undetectable using the applied methods. However, during heat treatment, the increased temperature could cause these low-concentration compounds to volatilize, resulting in their accumulation and detection in the final EPB.

A further Venn analysis of the 45 VOCs (Figure 1b) revealed that *Eucalyptus* wood contributed 28 compounds, including 25 terpenes and terpenoids and 3 aldehydes. *Pine* wood contributed 23 compounds, comprising 15 terpenoids and derivatives, 6 aldehydes, and 2 alkanes. Miscellaneous wood contributed 37 compounds, including 30 terpenes and terpenoids, 6 aldehydes, and 1 furan. Among these, 12 compounds were common to all three raw materials, 10 were shared between *Eucalyptus* wood and miscellaneous wood, and 9 were shared between *Eucalyptus* wood and *Pine* wood. Despite the relatively low proportion of miscellaneous wood in the EPB, it contributed the highest number of unique VOCs, with six compounds exclusive to this raw wood material. This suggests that miscellaneous wood may significantly impact the variety of VOCs in the final board. To control the diversity of VOCs, reducing the amount of miscellaneous wood in the formulation could help limit the contribution of its unique compounds. *Pine* wood, comprising approximately 24% of the EPB, contributed fewer unique compounds, with most of its VOCs similar to those from *Eucalyptus* wood. This indicates that *Pine* wood has a more limited impact on VOC composition, and adjusting its proportion in the production process could help optimize VOC types. Moreover, although eight compounds were detected in the additives (emulsions, auxiliaries, and adhesives), five of these were also found in the raw wood materials. The remaining three compounds, including eicosane and heneicosane, both high-boiling-point substances with boiling points above 300 °C, and tetracosane, a MVOC with a boiling point of 242.9 °C, suggest that most LVOCs in the additives likely evaporated during processing, which helps ensure that the final EPB meets environmental standards.

The VOC composition of EPB is predominantly made up of MVOCs and LVOCs, particularly terpenoids and derivatives, with sesquiterpenoids like aromadendrene, viridiflorol, and ledene being the most abundant. While these compounds contribute to the fragrance of the EPB and have various biological activities, high concentrations or prolonged exposure may cause irritation, especially under high temperatures. *Eucalyptus* wood, *Pine* wood, and miscellaneous woods serve as the main sources of VOCs, with new compounds also forming during the manufacturing process. Although GC-MS has been used to identify and quantify the components and their relative average concentrations in EPB, the contribution of each VOC to the overall odor is not directly proportional to its concentration. Therefore, a systematic and scientific approach is required to analyze and evaluate the key aromatic components that significantly influence the odor of EPBS.

### 2.2. Analysis of KOCs

**Figure 2:**
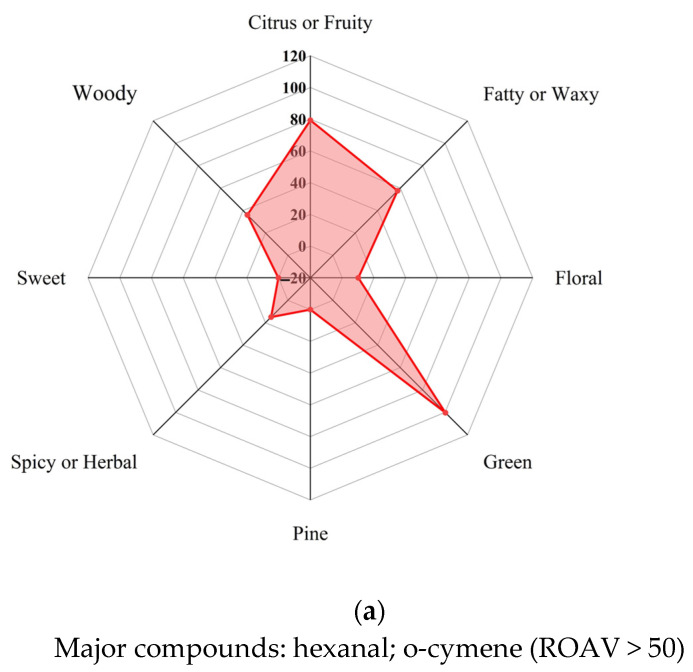

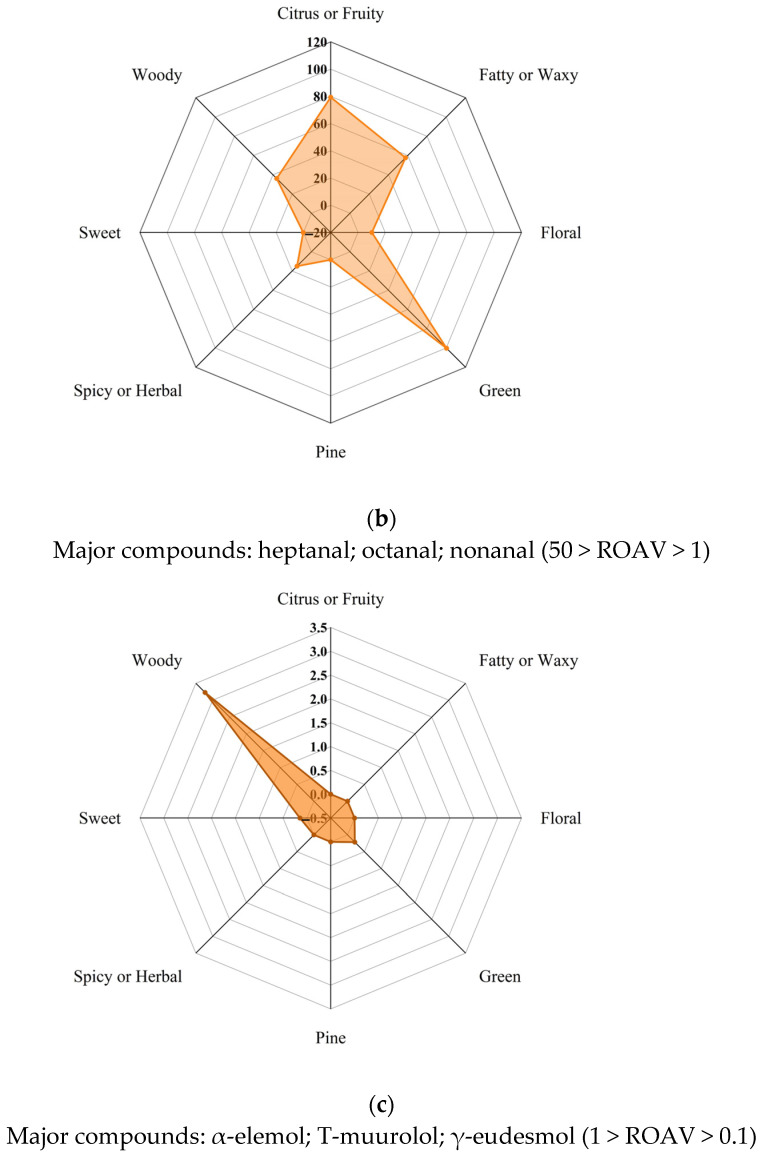
Odor radar charts based on different VOCs: (**a**) all VOCs; (**b**) VOCs with ROAV ≥ 1; (**c**) VOCs with 0.1 ≤ ROAV < 1.

Although over 10,000 odor compounds have been identified to date, only a small portion of these compounds play a decisive role in the odor of EPB. These compounds, referred to as KOCs, are crucial for understanding and optimizing the odor characteristics of EPB. The odor of EPB is not directly correlated with the relative content of VOCs but is better evaluated using the ROAV. Based on the GC-MS detection of VOCs and the OT of various compounds in air from the relevant literature, the ROAVs of 65 VOCs were calculated (Table S1). By summing the ROAVs of compounds with similar odor properties, radar charts for all VOCs, VOCs with ROAV ≥ 1, and VOCs with 0.1 ≤ ROAV < 1 were constructed. As shown in Figure 2, the overall odor profile of the 65 VOCs detected in EPB primarily features green, citrus or fruity, fatty or waxy, and woody characteristics, with subtle nuances of spicy or herbal, floral, sweet, and pine-like notes (Figure 2a). Compounds with ROAV ≥ 1 are typically defined as KOCs and play a key role in determining the overall odor. The results in Figure 2b strongly confirm this, showing that the 16 detected KOCs exhibit odor characteristics that are entirely consistent with the overall odor profile. In contrast, the 42 compounds with 0.1 ≤ ROAV < 1, although primarily woody in odor, have minimal impact on the overall odor characteristics.

A detailed analysis of the 16 KOCs reveals that they include 9 terpenoids and derivatives, 6 aldehydes, and 1 phenylpropanoid, primarily derived from raw materials such as *Eucalyptus* wood, *Pine* wood, and miscellaneous wood, as well as the production process. Among these compounds, the aldehyde hexanal has the highest ROAV, despite its relative content being only 1.67% [34]. With an OT value of just 0.0011 mg/m^3^ in air, its ROAV is 100. Its odor profile is characterized as green, grassy, and fresh, making it the most significant contributor to the odor of EPB. Next is the terpenoid o-cymene, with a relative content of 4.67%. Although its content is not high, its OT value is only 0.0040 mg/m^3^, resulting in an ROAV of 76.90. Its odor profile includes citrus, woody, and slightly sweet notes, making it another crucial compound contributing to the overall odor. Another aldehyde, heptanal, has a relative content of 0.27% but an ROAV of 20.92. Its odor profile includes fatty, green, and citrus notes, which also significantly contribute to the overall odor of EPB. Notably, hexanal has a boiling point of 128.8 °C, o-cymene has a boiling point of 186.3 °C, and heptanal has a boiling point of 151.9 °C. These three compounds are all volatile and mainly originate from the wood raw materials, with o-cymene being specific to *Eucalyptus* wood. Additionally, two other volatile aldehydes, octanal (relative content 0.14%) and nonanal (0.43%), with boiling points of 168.5 °C and 185.0 °C, respectively, are present. The odor profile of octanal is characterized as fatty, green, and citrusy, with an ROAV of 17.73. Nonanal, with an ROAV of 14.16, has an odor profile that includes fatty, waxy, and citrusy notes. Both of these aldehydes are derived from *Pine* wood, miscellaneous wood, and additives. The terpene viridiflorol, with an ROAV of 20.50 and a high relative content of 15.56%, has a higher boiling point of 372.2 °C, making it an LVOC. Its odor profile is woody, herbal, and slightly balsamic, primarily originating from *Eucalyptus* wood and miscellaneous wood. Safrole, possibly formed during the EPB production process, is a phenylpropanoid compound with a relative content of 0.07%, a boiling point of 231.7 °C, and moderate volatility. It has an ROAV of 13.17 and an odor profile characterized as spicy, warm, and sweet. The remaining nine compounds include terpenoids and derivatives as well as aldehydes. Six of these compounds are moderately volatile, and three are LVOCs, all of which play important roles in the overall odor of the EPB.

Further analysis of all VOCs reveals that, except for furfural, all detected aldehydes have ROAVs exceeding 4.5. Hexanal, with the highest ROAV among all compounds, indicates that aldehydes are the key contributors to the overall odor of EPB and can serve as representative compounds for evaluating its odor characteristics. Furthermore, the Eucalyptus-specific o-cymene also makes a significant contribution to the odor and can be considered a characteristic compound for assessing the odor of EPB.

Therefore, several strategies can be employed to optimize the odor quality of EPB. One effective approach is reducing HVOCs through controlled heating during post-processing, which can mitigate their influence. Additionally, limiting the use of miscellaneous wood, which introduces undesirable odors, can enhance the overall scent profile of the product. Finally, genetically modifying *Eucalyptus* to develop low-o-cymene-emitting varieties that produce fewer compounds with low OTs and high concentrations represents a promising method for improving raw material quality and enhancing the odor characteristics of EPB.

### 2.3. Comparison of VOCs After Thermal Post-Treatment Under Different Conditions

**Figure 3:**
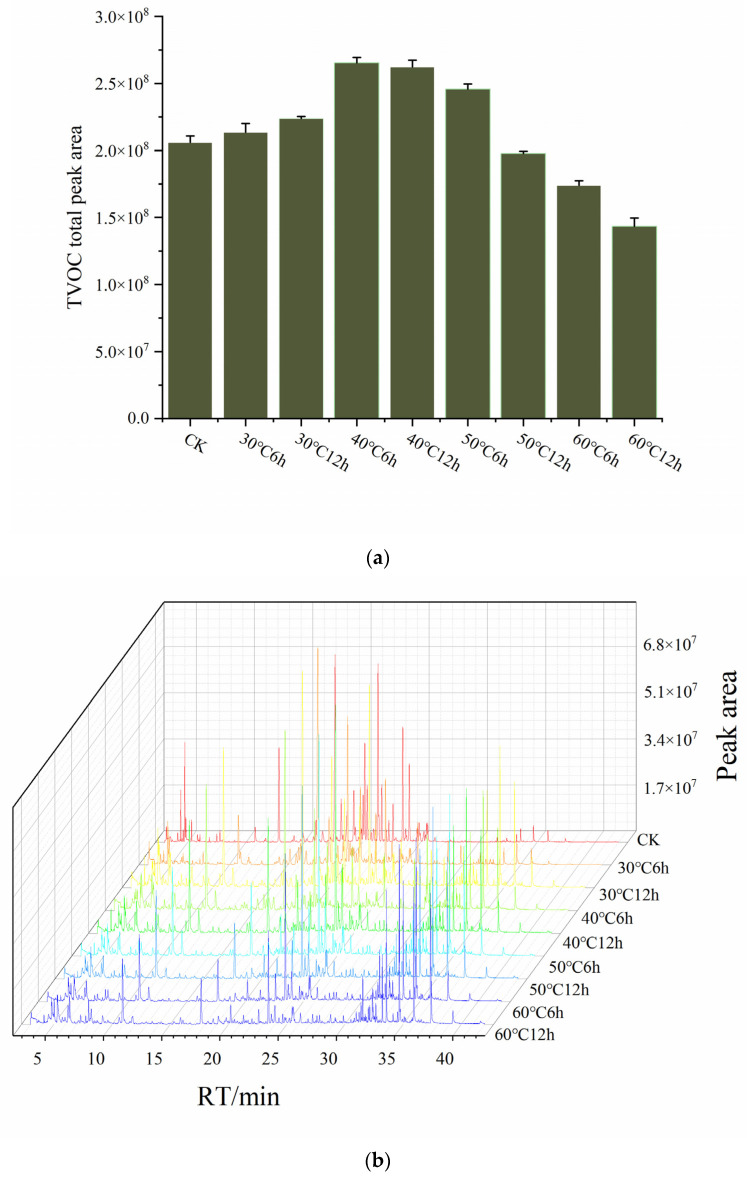

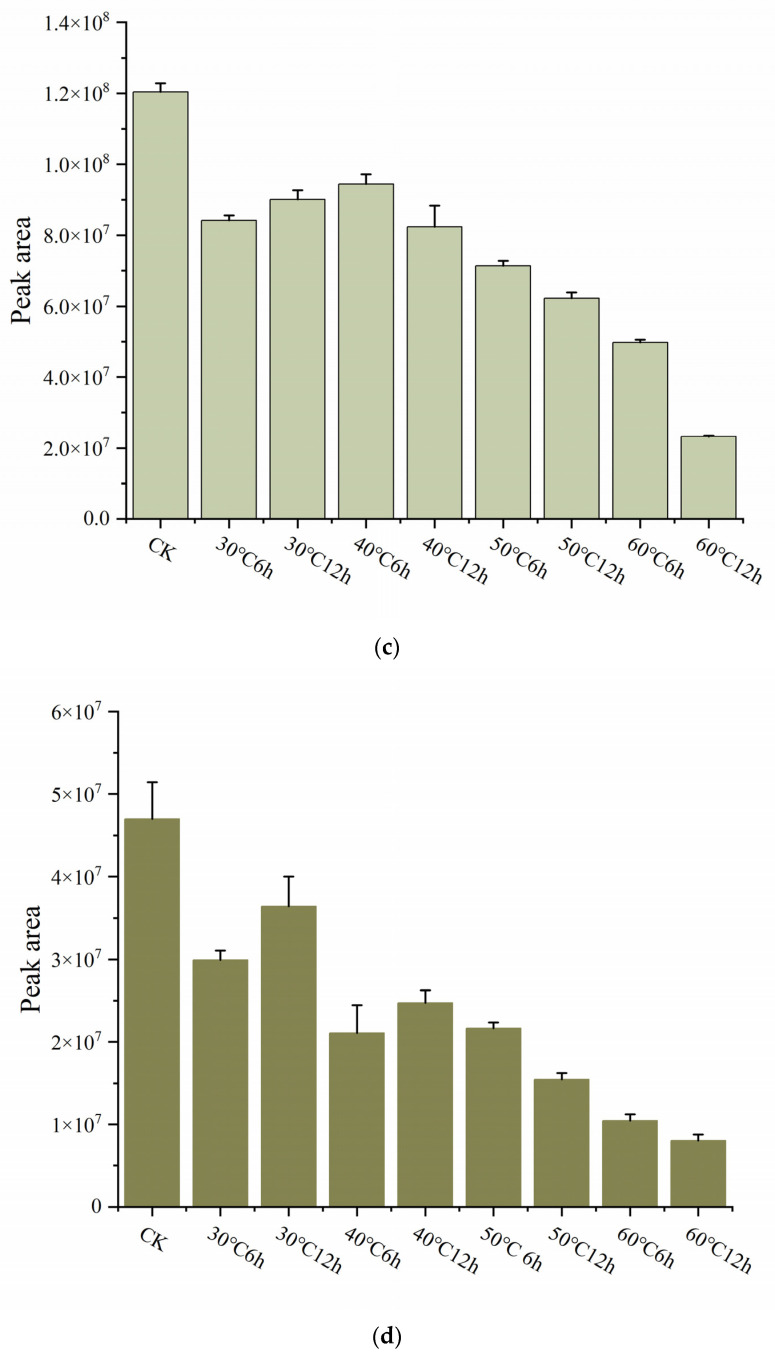
VOC analysis of *Eucalyptus* particleboard after thermal treatment under various conditions. (**a**) Total ion chromatograms of VOCs; (**b**) VOC peak areas; (**c**) peak areas of VOCs with ROAV ≥ 1; (**d**) peak areas of VOCs with 0.1 ≤ ROAV < 1.

Based on the analysis of VOCs and KOCs in EPB, it was found that the compounds remaining in the board after high-temperature pressing primarily consist of MVOCs, with smaller quantities of HVOCs and LVOCs. Among the HVOCs, most are classified as KOCs, which play a critical and decisive role in defining the overall odor characteristics of the board. Considering these characteristics, it can be inferred that appropriate thermal post-treatment processes, applied without compromising the structural and mechanical properties of the board, can effectively reduce the residual HVOCs and significantly lower the MVOC content. This can substantially improve the odor profile of board, providing important support for enhancing its quality and suitability for indoor applications. To explore this optimization strategy, EPBs were subjected to thermal post-treatments at 30, 40, 50, and 60 °C for durations of 6 and 12 h. The total VOCs, KOCs, and compounds with 0.1 ≤ ROAV < 1 under different treatment conditions were subsequently analyzed, with the results presented in Figure 3 and Table S2.

The analysis indicates that thermal post-treatments result in significant changes in the residual VOCs in EPB. As the temperature increases and the treatment duration extends, the peak area of residual VOCs initially increases and then decreases. Notably, after treatments at 30, 40, and 50 °C, the residual VOC content in the EPB is consistently higher than that in the untreated group. This suggests that even during storage, substantial amounts of VOCs continue to be released from the boards, potentially affecting the surrounding air quality. Therefore, accelerating the release of these odor-causing compounds through appropriate treatment before the boards are sold is crucial. Such measures can not only improve the olfactory characteristics of the EPB but also mitigate its impact on indoor air quality [15]. Analysis of the VOC residues in EPB under different thermal post-treatment conditions revealed that at 30 °C with a short treatment duration (6 h), only a small amount of VOCs were released, likely dominated by HVOCs. However, during the release process, some compounds may have been adsorbed or retained within the micropores of the wood structure, resulting in a slight increase in the VOC peak area compared to untreated boards. As the treatment duration was extended to 12 h, MVOCs and LVOCs began to volatilize gradually. However, due to the relatively low temperature, these compounds could not fully dissipate and instead accumulated within the surface or internal voids of the particleboard, leading to an observed increase in residual VOC content. When the temperature was raised to 40 °C and applied for 6 h, thermal energy accelerated the release of HVOCs and some MVOCs. However, certain compounds may have been adsorbed or retained within the microstructures of the wood during this process, causing the residual VOC peak area to increase. With an extended treatment time of 12 h, some volatile compounds gradually diffused to the surface of the particleboard and were released into the air, leading to a reduction in the residual VOC content. At 60 °C, with the treatment time extended, the thermal process significantly enhanced the diffusion and volatilization of VOCs within the particleboard, resulting in a continuous reduction in the overall residual VOC amount.

Furthermore, the analysis of KOCs with ROAV ≥ 1 in EPB subjected to different thermal post-treatments (see Figure 3c) shows that the peak area changes in KOCs follow a trend similar to those of total VOCs. However, the residual KOCs in the thermally treated boards are significantly lower than those in the untreated group. This can be attributed to specific compounds in the KOCs, such as hexanal, o-cymene, heptanal, octanal, and nonanal, which have high ROAVs and are classified as HMOVs. Thermal post-treatment accelerates the diffusion of these compounds from the interior of the board to the surface, promoting their volatilization and effectively reducing the residual KOCs. This process alleviates the odor impact of the board. Additionally, appropriate heating treatment can further accelerate the diffusion of these compounds from the inner layers to the surface, facilitating their evaporation and significantly reducing the residual KOCs, thus mitigating the odor impact of the particleboard [42,43].

### 2.4. Mechanisms of VOC Removal Through Thermal Post-Treatment

**Figure 4:**
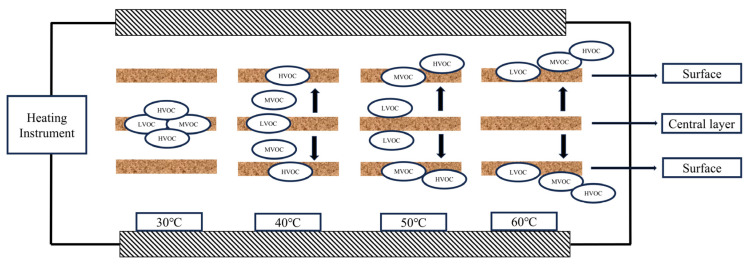
Mechanisms of VOC removal through thermal post-treatment. (↑/↓: VOC pathways; →: layered structure).

Based on the analysis of residual VOCs in *Eucalyptus* particleboard after post-treatment at different temperatures, we propose a process for the release of VOCs, as shown in the Figure 4.

The inherent porous structure of wood provides diffusion channels for VOCs while also potentially delaying the release of some low-volatility organic compounds through physical adsorption or retention [44]. Therefore, during the pressing process, although high temperatures cause a significant amount of VOCs to be released from the board, some VOCs remain trapped in the pores of wood or within the compacted material. Subsequent thermal treatment helps release the trapped VOCs. However, the release of these VOCs is influenced by factors such as material structure, molecular size, and volatility, leading to varying results under different treatment conditions.

The results indicate that temperature plays a critical role in the diffusion behavior and separation characteristics of VOCs. As temperature increases, the thermal motion of VOCs intensifies, accelerating their migration from the interior of the material to the surface, thereby increasing the diffusion coefficient [45,46]. This increase in the diffusion coefficient directly leads to a faster release of VOCs into the air, gradually reducing the residual VOCs within the material. Moreover, higher temperatures elevate the saturation vapor pressure of VOCs, making them more likely to desorb from the surface of the material into the gas phase. This phenomenon decreases the partition coefficient between the solid and gas phases, further accelerating the escape rate of VOCs. As the separation process intensifies, the release rate of VOCs increases, leading to a significant reduction in residual VOCs in *Eucalyptus* particleboard [47].

## 3. Discussion

Based on these research findings, we propose the following recommendations to reduce VOCs in EPB:

(1) Minimize the use of miscellaneous woods with strong odors: The inclusion of miscellaneous woods with strong odors increases the diversity of VOCs emitted. It is recommended to reduce or eliminate the use of miscellaneous woods in the production of EPB to mitigate odor. If the use of miscellaneous wood is unavoidable in the production process, it is recommended to implement baking or heat treatment to minimize the release of VOCs and odor emissions.

(2) Control of o-cymene: o-Cymene, an LVOC detected exclusively in *Eucalyptus* wood raw materials, has a high ROAV of 76.90, significantly contributing to the overall odor. Therefore, it is advisable to explore the biosynthetic pathway of o-cymene in *Eucalyptus* from a forestry genetic breeding perspective. Genetic modifications can be conducted to develop *Eucalyptus* varieties with lower o-cymene content or eliminate its production entirely.

(3) Post-treatment temperature control: If improvements cannot be made at the raw material level, it is recommended to reduce the temperature to 50–60 °C during the cooling process after pressing (keeping the temperature as high as possible without affecting the basic properties of the board). This temperature should be maintained for a certain period. Additionally, exhaust ventilation systems should be installed to promptly release VOCs, which will help reduce residual VOCs in the EPB and consequently lower the odor concentration.

(4) Usage recommendations for consumers: For consumers using EPB, it is advisable to keep the material away from heat sources and ensure adequate ventilation during use.

Suggestions for enterprise production: It is recommended to install odor control systems to protect workers in case such emissions occur due to high heat. To effectively reduce the concentration of VOCs in production workshops, enterprises must adopt comprehensive measures encompassing source control, process management, end-of-pipe treatment, and ongoing monitoring. Specific measures are presented in Table S3.

(5) Government implementation of pertinent measures: It is recommended to expedite the establishment of an environmental regulatory framework centered on the control of VOCs and odorous gases. This framework should incorporate regulatory measures such as formulating graded emission standards, implementing full-process online monitoring systems, and developing industry-specific best available technology (BAT) guidelines to mandate cleaner production upgrades in enterprises. Concurrently, a dedicated fund for environmental technology retrofits should be established to provide tax credits and equipment subsidies for enterprises adopting high-efficiency treatment technologies (e.g., adsorption recovery and catalytic combustion). Such integrated policies will facilitate the transition of industrial production toward green and low-carbon practices that achieve dual benefits in environmental performance and economic competitiveness. Specific measures are presented in Table S4.

## 4. Materials and Methods

### 4.1. Materials

The EPB (thickness scale: 18 mm, supplied by Kunming Feilin Artificial Board Group Co., Ltd., Kunming, China), consisted of approximately 75% *Eucalyptus* wood, 24% *Pine* wood, and 1% miscellaneous wood. It was bonded using urea–formaldehyde resin (molar ratio: 1.0; viscosity: 20–25 s (s); relative resin content: 100–105 kg/m^3^ (constituting approximately 13%) and continuously pressed under hot-pressing conditions at 180–240 °C and 1–4 MPa. For basic information regarding its basic physical and mechanical properties, please refer to Table S5. Before experimentation, the EPB was processed using a high-speed multifunctional crusher (Giant Hongfeng JHF-1000A, supplied by Kunming Tieshen Trading Co., Ltd., Kunming, China). The resulting material was then sieved through a 40-mesh sieve and uniformly mixed before being sealed for later use.

### 4.2. Thermal Post-Treatment

First, 10 g of crushed and sieved EPB particles were accurately weighed and evenly distributed into a glass dish with a 5 cm diameter. The dish was placed in a preheated 101-2ES electric blast drying oven (101-2ES, supplied by Beijing Yongguangming Medical Instrument Co., Ltd., Beijing, China) set to various temperatures (30, 40, 50, and 60 °C), and the conditions were maintained for 6 and 12 h. A total of 27 EPB samples were prepared, and their group assignments are listed in Table 1.

### 4.3. Enrichment of VOCs and Component Analysis

After treatment under different temperatures, 1 g samples were quickly transferred to 50 mL headspace vials. Then, the samples were equilibrated at 90 °C for 34 min to ensure a uniform distribution of the VOCs released by the samples within the vials. Next, a 75 μm CAR/PDMS solid-phase microextraction (SPME) head (57344-U, Supelco, Bellefonte, PA, USA) that had been preconditioned (held at 250 °C for 2 h until no interference peaks were observed) was inserted into the vial for VOC enrichment for 24 min. Once the extraction was complete, the SPME head was rapidly inserted into the manual injection port of the GC-MS instrument (7090B/5977B, Agilent, Santa Clara, CA, USA) for thermal desorption and analysis. During the injection process, the chromatographic system was operated without split flow. Helium was used as the carrier gas, and an HP-5MS quartz capillary column (30 m × 250 μm × 0.25 μm, Shiseido Co., Ltd., Tokyo, Japan) was employed. The injection port temperature was set to 300 °C. The temperature program began at 60 °C, increased at a rate of 10 °C/min to 120 °C, was held for 10 min, then increased by 5 °C/min to 180 °C, and finally increased by 10 °C/min to 240 °C and was held for 8 min. The MS system used an electron impact ionization (EI) source with an electron energy of 70 eV. The transfer line temperature was set to 250 °C, and the ionization source temperature was set to 230 °C, while the quadrupole was held at 150 °C. Data were collected in full-scan mode, with the mass scan range set from 35 to 550 *m*/*z*. The NIST14 standard spectral library was used for computer retrieval and qualitative analysis, with target peaks integrated and calibrated while excluding interference from non-target ions. The relative content of each VOC was determined by area normalization, and VOCs with match scores greater than 80 (out of a maximum of 100) were subjected to qualitative analysis [48,49].

### 4.4. Analysis of KOCs

Following the methods proposed by Zhu Yifan et al. [50] and Lan Yibin et al. [51], with slight modifications, the VOCs detected in the samples were cross-referenced with their odor threshold (OT) values in air, as reported in the Compilation of Odor Thresholds for Compounds, 2nd Edition [52]. For compounds not listed in this reference, their OT values were predicted based on their physicochemical properties, molecular structural features, volatility, known olfactory characteristics of similar compounds, and their relationships with olfactory perception mechanisms. The relative odor activity value (*ROAV*) method was then applied to identify the key odor-contributing compounds (KOCs) in EPB. The odor activity value (*OAV*) and *ROAV* were calculated using Equations (1) and (2), respectively.

(1) $${OAV}_{i}=\frac{C_{i}}{T_{i}}$$

(2) $$ROAV=\frac{{OAV}_{i}}{{OAV}_{m}}\times 100$$

where $OAV_{i}$ represents the odor activity value of volatile compound *i*. $C_{i}$ denotes the relative content (%) of volatile compound *i*, and ${OT}_{i}$ represents the odor threshold (in air, mg/m^3^) of volatile compound *i*. $C_{max}$ and $T_{min}$ represent the relative content (%) and the corresponding odor threshold (in air, mg/m^3^) of the volatile compound contributing most significantly to the overall odor of the sample.

Typically, VOCs with ROAV ≥ 1 are considered KOCs, while those with 0.1 ≤ ROAV < 1 are deemed to have a significant influence on the overall odor. The VOC with the highest contribution to overall odor of the sample is defined as having *ROAV* = 100. Furthermore, VOCs with *ROAV* ≥ 1 are identified as key odor contributors in the sample. By combining the odor characteristics and ROAV values of the VOCs, the *ROAVs* of VOCs with similar odor characteristics were summed, and an odor radar chart was constructed to visualize the distribution of the odor profile of sample.

### 4.5. Data Processing

Data were processed using Microsoft Excel 2019 and IBM SPSS Statistics 26. GraphPad Prism 8 was used to generate extraction head CANV plots, while total ion chromatogram analyses were visualized with Origin 2022.

## 5. Conclusions

The VOC mixture released from EPB exhibits a complex chemical composition. A total of 65 different compounds were identified through HS-SPME coupled with GC-MS analysis. The residual compounds in the EPB after high-temperature treatment were primarily MVOCs, with smaller amounts of HVOCs and LVOCs. These results indicate that terpenoids are the predominant volatile components released from the EPB, accounting for 78.46% of the total VOCs. Aldehydes, although representing only 10.77% of the total, contribute significantly to the odor of the particleboard, with low-OT compounds such as hexanal and pentanal identified as key contributors to the overall emission profile. Notably, although only 1% of miscellaneous wood was incorporated into the particleboard, 37 VOCs were derived from it. This suggests that the quantity and type of wood materials used can have a substantial impact on the VOC release characteristics of the particleboard. Thermal post-treatment proves effective in reducing the release of both total VOCs and odors from the particleboard. However, the effectiveness of this process is closely linked to treatment temperature and duration. Lower-temperature treatments (30 °C or 40 °C) did not significantly reduce VOC release, indicating that the emission of VOCs from the particleboard is a continuous process, with most VOCs having relatively high boiling points. In contrast, thermal treatment at moderate temperatures (such as 50 °C or 60 °C) resulted in a marked reduction in both VOC and odor emissions. Therefore, thermal post-treatment at moderate temperatures is an effective method for reducing VOCs and odor emissions from particleboards and other composite wood materials. Based on considerations of economic feasibility, environmental impact, and factory safety, treatment at 60 °C for 6 h proves to be an effective and safe approach to controlling VOC and odor release from wood-based products.

This study innovatively integrates HS-SPME coupled with GC-MS and odor threshold theory from food science, establishing a quantitative odor intensity evaluation method based on OAVs. This approach precisely and rapidly identifies key odor-active compounds in particleboard, including low-OT substances such as hexanal and pentanal. Compared to conventional physical encapsulation or chemical spraying methods (e.g., VOC-degrading agents), the proposed moderate-temperature (50–60 °C) post-treatment process achieves significant VOC residue elimination within 6 h without secondary contamination risks. The technology effectively reduces overall odor intensity below human perception thresholds by disrupting the gas–solid equilibrium of high-boiling-point terpenoids, thereby addressing consumer concerns about long-term VOC emissions from wood-based building materials.

## Figures and Tables

**Table 1:** Group assignments of 27 samples and their respective categories.

No.	Sample Name	Group	Sample Information
1	CK-1	Untreated	Blank control
2	CK-2		
3	CK-3		
4	30 °C 6 h-1	30 °C 6 h	Heated at 30 °C for 6 h
5	30 °C 6 h-2		
6	30 °C 6 h-3		
7	30 °C 12 h-1	30 °C 12 h	Heated at 30 °C for 12 h
8	30 °C 12 h-2		
9	30 °C 12 h-3		
10	40 °C 6 h-1	40 °C 6 h	Heated at 40 °C for 6 h
11	40 °C 6 h-2		
12	40 °C 6 h-3		
13	40 °C 12 h-1	40 °C 12 h	Heated at 40 °C for 12 h
14	40 °C 12 h-2		
15	40 °C 12 h-3		
16	50 °C 6 h-1	50 °C 6 h	Heated at 50 °C for 6 h
17	50 °C 6 h-2		
18	50 °C 6 h-3		
19	50 °C 12 h-1	50 °C 12 h	Heated at 50 °C for 12 h
20	50 °C 12 h-2		
21	50 °C 12 h-3		
22	60 °C 6 h-1	60 °C 6 h	Heated at 60 °C for 6 h
23	60 °C 6 h-2		
24	60 °C 6 h-3		
25	60 °C 12 h-1	60 °C 12 h	Heated at 60 °C for 12 h
26	60 °C 12 h-2		
27	60 °C 12 h-3		

## Data Availability

The data that support the findings of this study are available from the corresponding author upon reasonable request.

## Supplementary Materials

The following supporting information can be downloaded at: https://www.mdpi.com/article/10.3390/molecules30091949/s1, Table S1. Characteristics of VOCs Identified in EPB by HS-SPME Coupled with GC-MS. Table S2. VOC Emission Characteristics of EPB under Different Thermal Post-Treatment Conditions. Table S3. Classification of common technologies for VOC and odor control. Table S4. Direct restrictions of odor in different country and region. Table S5. Fundamental basic regarding the basic physical and mechanical properties of *Eucalyptus* wood particleboard. Figure S1. Ion chromatogram. Refs. [53,54] are cited in Supplementary Materials.
