# Supplementary material for: Unlocking the Potential of Thermal Post-Treatments: A Study on Odor Emission Control in Eucalyptus Wood Particleboard
Source: Molecules. 2025 Apr 28;30(9):1949. doi: 10.3390/molecules30091949 (PMC12073479; doi:10.3390/molecules30091949)
Supplement: Supplementary file 1 [file molecules-30-01949-s001.zip › TableS3-S5; Figure S1.pdf]

Supplementary materials

Table S3 Classification of common technologies for VOC and odor control [1]

Physical	Chemical	Biological
Condensation	Combustion	Biofiltration
Membranes	Chemical precipitation	Biotrickling flters
Masking	Plasma technology	Bioscrubbers
Dilution	Oxidation	Membrane bioreactors
Absorption	Others	Phctochemical bioreactors
Adsorption		Rotating Biological Contactors
Others		Suspended cell bioreactor
		Others

Table S4 Direct restrictions of odor in different country and region[2]

	Nuisance	Site of required compliance		D/T(dilutions to hreshold) [a]	Chemical-specific [b]
		Off-site receptor	Property boundary		
Japan	Yes	Yes	Yes	Yes	Yes
Peoples Republic of China	Yes	Yes			
United Kingdom	Yes	Yes			
Canada					
Ontario	Yes	Yes			Yes
Quebec	Yes	Yes		Yes	
United States of America					
New York	Yes	Yes			
Michigan	Yes	Yes			
Massachusetts	Yes	Yes	Yes	Yes	Yes
Arkansas	Yes	Yes			

a Compliance level is measured in concentration of mixed chemicals whose odor first meets the odor threshold at a specific number of dilutions of an air sample of the odorous air.

b For instance, may include ammonia, amines, hydrogen sulfide, mercaptans, dimethyl disulfide, dimethyl sulfide, fatty acids, limonene, other terpene compounds.

Table S5 Fundamental basic regarding the basic physical and mechanical properties of *Eucalyptus* wood particleboard

Mechanical characteristics	Value/Result	Test Standard
Density (g/cm^3)	0.68	ISO 9427
Thickness Swelling (%)	1.41	EN 317
MOE (MPa)	15.32	EN 310
MOR (MPa)	2980	EN 310
IB Strength (MPa)	0.51	EN 319
Formaldehyde (mg/100g)	2.30	EN 120 (Perforator)
Formaldehyde (mg/L)	0.44	JIS A 1460 (Desiccator)

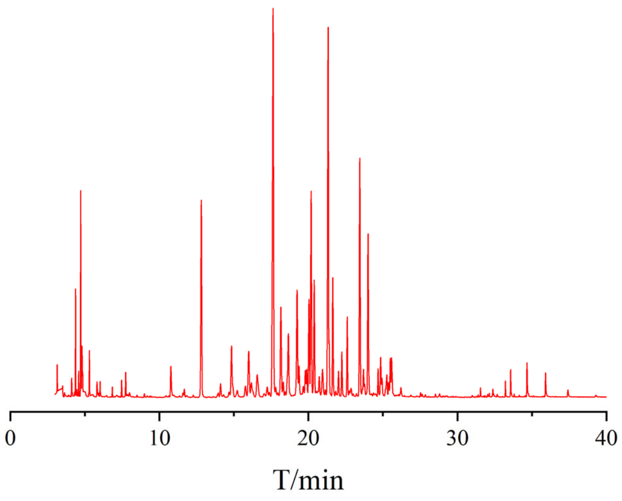

Figure S1 Ion chromatogram

References

[1] Revah, S. Morgan-Sagastume, J.M. Methods of Odor and VOC Control. Springer Berlin Heidelberg, 2005. DOI: 10.1007/3-540-27007-8_3.

[2] Aldrich, R.L. Environmental Laws and Regulations Related to Odor and Waste Gas Contaminants. Springer Berlin Heidelberg, 2005. DOI:10.1007/3-540-27007-8_2.
